# Non-communicable diseases attributed mortality and associated sociodemographic factors in Papua New Guinea: Evidence from the Comprehensive Health and Epidemiological Surveillance System

**DOI:** 10.1371/journal.pgph.0000118

**Published:** 2022-03-25

**Authors:** Bang Nguyen Pham, Ronny Jorry, Nora Abori, Vinson D. Silas, Anthony D. Okely, William Pomat

**Affiliations:** 1 Papua New Guinea Institute of Medical Research, Goroka, Papua New Guinea; 2 School of Health & Society and Early Start, University of Wollongong, Wollongong, Australia; Imperial College London, UNITED KINGDOM

## Abstract

**Background:**

Papua New Guinea (PNG) is undergoing an epidemiological transition with increased mortality from NCDs. This study examined NCDs-attributed mortality and associated sociodemographic factors in PNG.

**Method:**

Using WHO 2016 instrument, 926 verbal autopsy (VA) interviews were conducted in six major provinces from January 2018 to December 2020. InterVA-5 tool was used to assign causes of death (COD). Multivariable logistic regression analysis was performed to identify sociodemographic factors associated with mortalities from emerging and endemic NCDs.

**Finding:**

NCDs accounted for 47% of the total deaths, including 20% of deaths attributed to emerging NCDs and 27% of deaths due to endemic NCDs. Leading CODs from emerging NCDs were identified including cardiac diseases, stroke, and diabetes. The risk of dying from emerging NCDs was significantly lower among populations under age 44y compared with population aged 75+y (OR: 0.14 [0.045–0.433]; p-value: 0.001). People living in urban areas were twice likely to die from emerging NCDs than those in rural areas (OR: 1.92 [1.116–3.31]; p-value: 0.018). People in Madang province were 70% less likely to die from emerging NCDs compared to those from East New Britain province (OR: 0.314 [0.135–0.73]; p-value: 0.007). Leading CODs from endemic NCDs included digestive neoplasms, respiratory neoplasms, and other neoplasms. Only children aged 0-4y had significant lower risk of dying from endemic NCDs compared to the population aged 75+y (OR: 0.114 [95% CI: 0.014–0.896]; p-value: 0.039).

**Conclusion:**

Public health interventions are urgently needed, prioritizing urban population and those aged over 44y to reduce premature mortality from NCDs.

## Introduction

Non-communicable diseases (NCDs) are medical conditions or diseases that are, by definition, non-infectious and non-transmissible between people [1]. NCDs are the leading cause of death (COD) and kill 41 million people each year, equivalent to 71% of all deaths globally. Sustainable Development Goal (SDG) 3.4 states: “By 2030, reduce by one third premature mortality from NCDs through prevention and treatment” [2]. The Global Burden of Disease Study showed that cardiovascular diseases, cancers, and chronic respiratory diseases were the main causes contributing to the global and regional premature mortality from NCDs [3].

There has been a change in the global distribution of the burden of NCDs. Compared to high-income countries, NCDs now disproportionately affect populations in low- and middle-income countries (LMICs) [4]. About 85% of ‘premature deaths’ between the ages of 30 and 70 years and 77% of all NCD deaths each year occur in LMICs [5]. The examination of NCDs in LMICs has only recently been a research priority. The highest risks of dying from NCDs were observed in LMICs, especially in sub-Saharan Africa, Central Asia and Eastern Europe [6]. This epidemiological shift provides a starting point for better understanding NCDs in LMICs [7]. Understanding the socioeconomic determinants of mortality and morbidity from NCDs among the working age population is important [8, 9]. Studies on socioeconomic factors of NCDs are often limited to analysis of the effect of income per capita on the level of mortality. The association between sociodemographic factors and NCDs has been well established in high-income countries, but these associations are less clear in LMICs [10]. Understanding sociodemographic factors associated with mortality attributed to NCDs is important for the development of public health policy and programming interventions to achieve SDG targets [11].

Papua New Guinea (PNG) has a very young population, with 38.2% of the population under the age of 15 years [12]. The country has been reported undergoing an epidemiological transition, characterised by a shift from infectious diseases to NCDs [13, 14]. The increase in the prevalence of NCDs has placed additional burden on the healthcare system, particularly at the primary health level [14, 15]. The PNG Government has set a target to reduce premature NCD mortality by 25% from 2010 to 2020 [16]. The increased prevalence of NCD associated risk factors in PNG is likely associated with recent changes in socioeconomic development [17, 18]. However, little evidence is available to support this argument. Previous studies have reported causes of death in PNG [19], but not on sociodemographic factors associated with NCDs mortality and patterns. How household socioeconomic and demographic factors have contributed to the emergence of NCDs, driving the current mortality transition in PNG is unclear. To what extent the mortality from NCDs is taking place across ages and sexes of the population, urban-rural sectors, provinces and social classes is also little known. Addressing this current knowledge gap would assist the PNG Government in restructuring the healthcare systems, and prioritizing healthcare services, potentially contributing to a reduction in premature NCDs mortality and further improvement in health and well-being among the population.

## Objective

This study aimed to identify sociodemographic factors associated with mortality from NCDs among the general population in PNG by analysing the association of sociodemographic characteristics of the deceased and their causes of deaths.

## Materials and methods

### Data source

Mortality surveillance data were extracted from the Comprehensive Health and Epidemiological Surveillance System (CHESS), which was designed as a population-based longitudinal study, and established by Papua New Guinea Institute of Medical Research (PNGIMR). CHESS covers both urban and rural populations living in eight sentinel surveillance sites in six major provinces of PNG: Central, Eastern Highlands, East Sepik, Madang, East New Britain (ENB) and Port Moresby (POM)—the National Capital District. These provinces represent the four geographical regions of PNG: Highlands, Southern, Momase and Islands. The selection of these sites was based on the previous integrated Health and Demographic Surveillance System (iHDSS), with adjustment for inclusion of new urban sites in consultation with national and local level stakeholders. The urban-rural sites were defined based on the National Statistics Office’s definition applied to the National Census 2010 [20]. CHESS covered a population of approximately 54,399 at the baseline, equivalent to 0.65% of the total population in PNG in 2018 [21, 22]. The age and sex structures of the surveillance population are similar to those of the entire population in PNG [23]. The design and methods of CHESS have been previously published [24, 25].

### Data collection

The mortality surveillance data were collected using the WHO 2016 verbal autopsy (VA) instrument [26]. The instrument is programmed for conducting VA interviews by using portable electronic devices such as smart phones, iPADs and tablets. This tool does not require interviewers to have medical background to conduct VA interviews. Hence, the tool offers considerable improvement in the implementation [27].

The WHO 2016 VA tool has been adapted for suitable use in PNG. Aside from asking standard questions about the signs and symptoms the deceased experienced prior to death, an additional data module on the deceased identification information was included in the questionnaire, including household location (GPS data) and individual background information. This information allow linkages between mortality data and other existing data components available from the CHESS database such as morbidity data and household socioeconomic data, to enhance the scope of data for analysing sociodemographic factors associated with NCDs mortality in PNG [18]. VA interviews were conducted by the CHESS demographic team to collect mortality data from the communities from January 2018 to December 2020.

### Analysing cause of death

The InterVA-5 diagnostic tool was used for cause of death (COD) analysis. This analytical tool is a computer-based programme that can assign 64 specific CODs and categories aligned with the International Classification of Diseases version 10 [28]. The programme successfully assigned CODs for 926 deaths. The specific ascribed CODs were then grouped into four main categories:

- Infectious diseases (corresponding to Group I of the Global Burden of Disease Study);
- Emerging NCDs include cardiac diseases (acute and others), stroke, diabetes;
- Endemic NCDs included neoplasms, chronic obstruct pulmonary disease, asthma, gastrointestinal disorders, renal disorders, mental and nervous system disorders, malnutrition and endocrine disorders (emerging and endemic NCDs correspond to Group II);
- Injuries and other external causes (corresponding to Group III) [19].

The rationale for grouping NCD-attributed CODs into emerging NCDs and endemic NDCs has been described elsewhere [14].

### Analysing sociodemographic factors

Sociodemographic analyses were conducted incorporating three steps. First, COD data were linked with household socioeconomic status (SES) data derived from CHESS database for the corresponding period of time. Data on CODs of 689 deceased were successfully linked with household SES data, using the unique household identification codes. No household SES data from East Sepik Province (ESP) were available as the site was only opened in 2019.

A new variable for household wealth index was then created for all COD records. Household wealth index was an overall marker of household SES. This variable was constructed based on household socioeconomic variables including housing characteristics, access to water and sanitation, and household assets, using the principal component analysis (PCA) method. Variables on marital status, education, relationship to the household head, employment status, and access to healthcare services were not statistically significant in the PCA models hence they were excluded from the statistical model. Household wealth indices were then divided into five quintiles, representing the poorest, poor, middle, richer and the richest quintile [23].

Finally, multinomial logistic regression (MLR) analyses were conducted to identify sociodemographic factors associated with mortalities from emerging and endemic NCDs. Since social determinants of mortalities from emerging NCDs and endemic NCDs could be different [13, 18], analyses were conducted separately for these two categories to predict the risks of dying from these COD categories across sub-populations. Two binary variables (Yes/No) on emerging NCDs and endemic NCDs attributed deaths were created in the dataset. These variables were included in MLR models as dependent outcome variables with deaths to other CODs being used as the reference category. Sociodemographic variables were used as independent factors.

Significant factors remained in the MLR model, including sex (male and female), age at death (grouped into 0–4, 5–14, 15–24, 25–34, 35–44, 45–54, 55–64, 65–74, and 75+ years), urban-rural sector, provinces, and household wealth quintiles, assuming other predictors remained constant. The main effect was selected in these MLR models to produce maximum likelihood estimates of odds ratios (OR) for mortalities attributed to emerging NCDs and endemic NCDs. The statistical likelihood test was selected to calculate 95% confidence intervals (CI) of OR estimates. A p-value of 0.05 was used to determine statistical significance. No correction for multiple testing was applied to the p-values in study. All the p-values, odds ratios and they were directly produced from the MLR models. Data analyses were performed using Statistical Package for Social Science (SPSS) (version 20) (see S1 Data).

### Ethics approval and informed consent

The CHESS was granted ethics approvals from Instructional Review Board of PNG Institute of Medical Research (IRB’s Approval no. 18.05) and the Medical Research Advisory Committee of Papua New Guinea (MRAC’s Approval no. 18.06). These approvals covered all the data components under the CHESS, including the mortality data which were used in this manuscript.

VA interviews were conducted with informed consent. Participant information and consent were integrated as part of the VA questionnaire of the WHO 2016 VA tool. They were informed about their right to withdraw from the study at any stage. Informed consent was sought from self-identified close relatives of the deceased. Informed consent was obtained verbally from all respondents preceding the VA interview. The consents were written in the tablets with name of participant, name of witness and date of consent. VA interviews were conducted at the respondent’s home unless otherwise requested, and in the witnesses of other family or community member. All VA interview respondents were matured adults with close family relationship to the deceased. Among the 1021 deaths identified by the data reporters in the communities through household visits, consents were obtained for conducing 1003 VA interviews, resulted in a participation rate of 98%.

## Results

**Fig 1:**
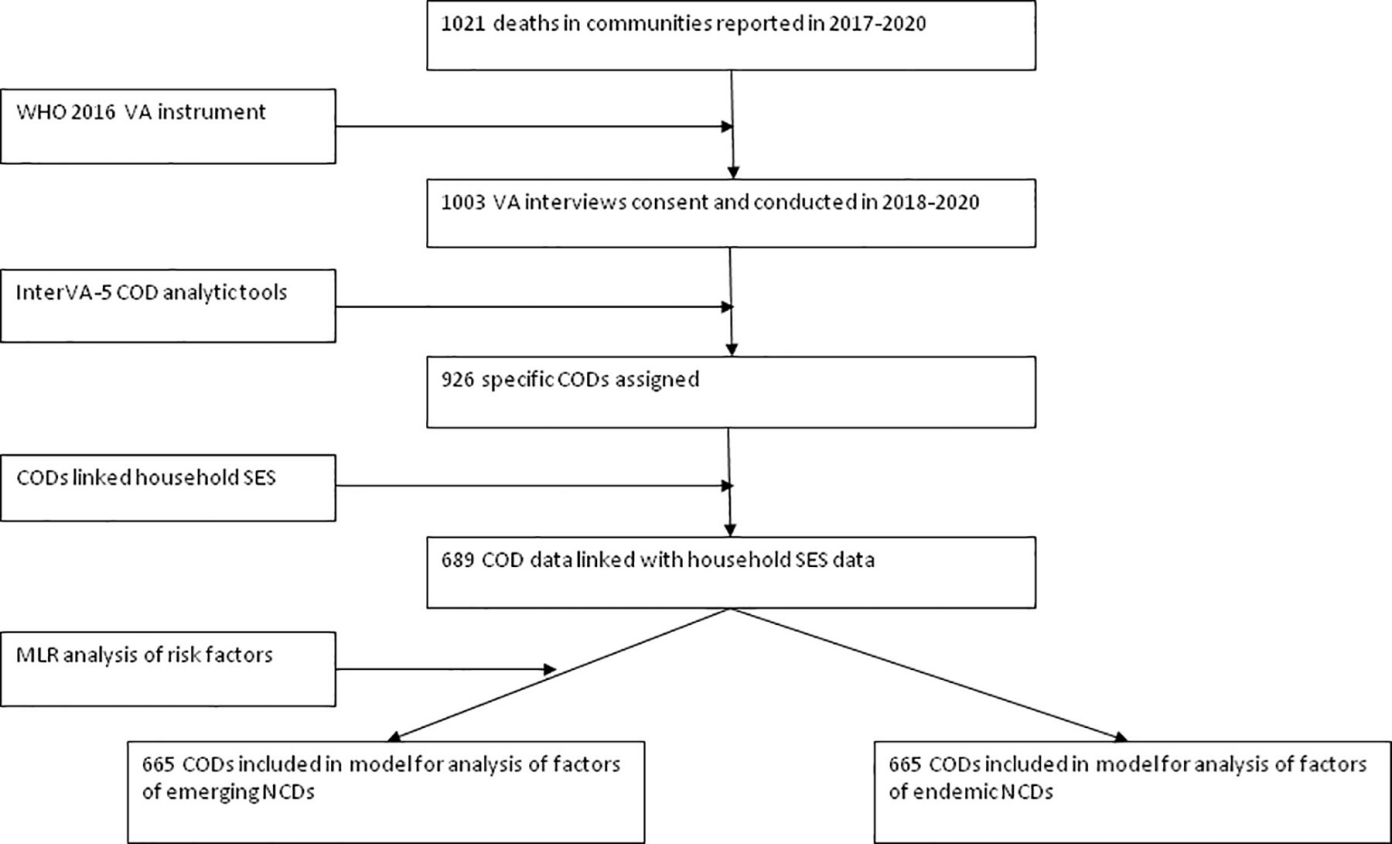
Flow chart for identification of deaths, selection for interview, and analyses of sociodemographic factors of mortalities from emerging and endemic NCDs in the population in PNG, PNGIMR’s CHESS, 2020.

Among the 1021 deaths in the communities that were identified by the CHESS’s data reporters based in the surveillance sites through household visits, consents were obtained for conducing 1003 VA interviews, resulted in a participation rate of 98%. The flow chart for death identification, selection for interview and data analysis is shown in Fig 1.

### Distribution of CODs

NCDs accounted for the largest proportion (47%) of the total deaths, including 20% of deaths attributed to emerging NCDs and 27% of deaths due to endemic NCDs. Infectious diseases accounted for the second largest proportion, 34% of the total deaths, followed by injuries and other external CODs (12%). Neonatal deaths and still births accounted for 3% and maternal deaths was 1%. About 3% of the deaths were unable to identify a specific COD.

### Leading causes of death from NCDs

**Fig 2:**
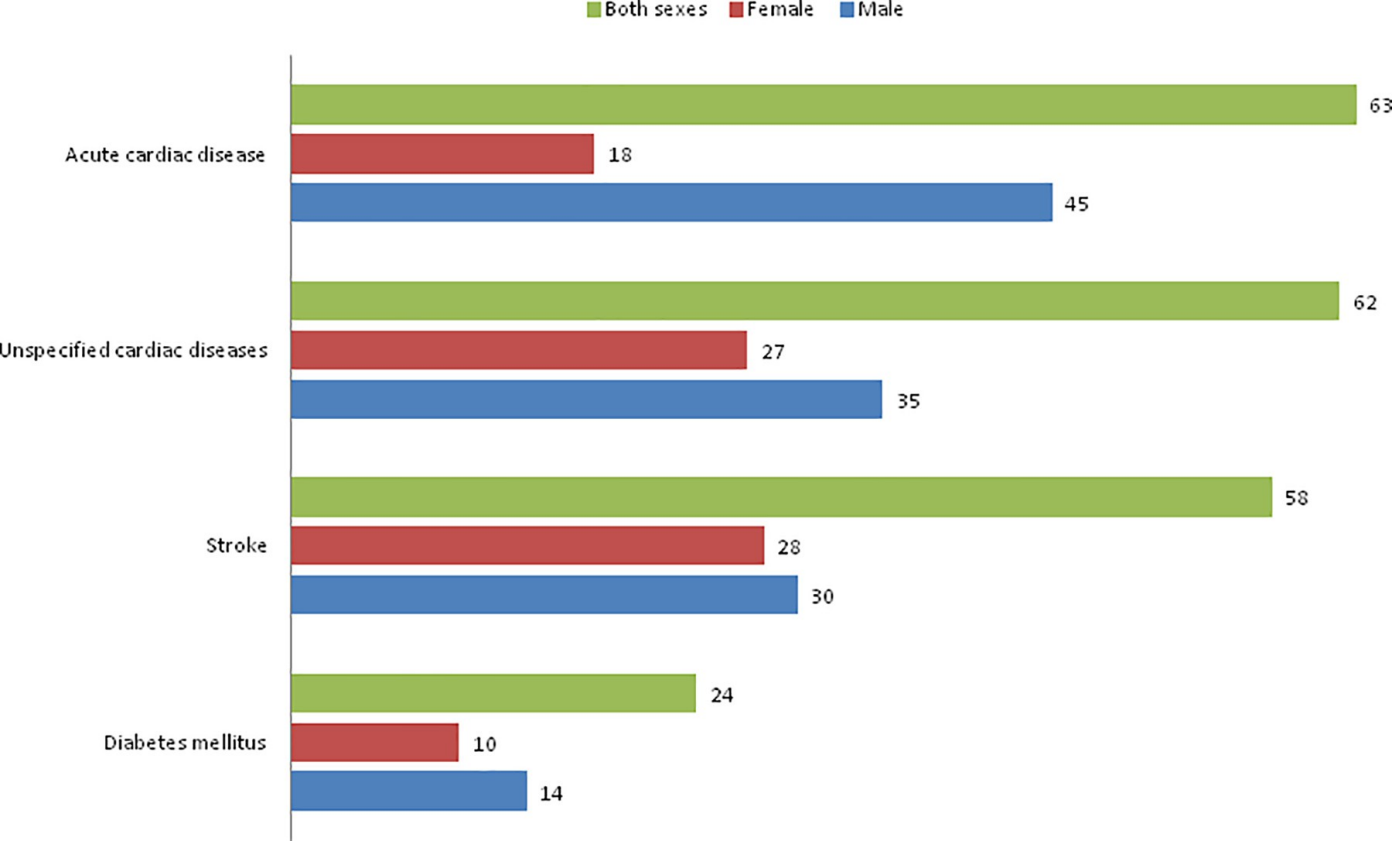
Numbers of deaths from emerging non-communicable diseases, PNGIMR’s CHESS, 2020.

Fig 2 shows the leading COD from emerging NCDs. Acute cardiac diseases (ACD) was identified as the first leading CODs, followed by unspecified cardiac diseases, stroke, and lastly diabetes. The numbers of male deaths to the emerging NCDs were slightly higher the female deaths, but the difference was not statistically significant (Chi-squared test: p-value at 0.086).

**Fig 3:**
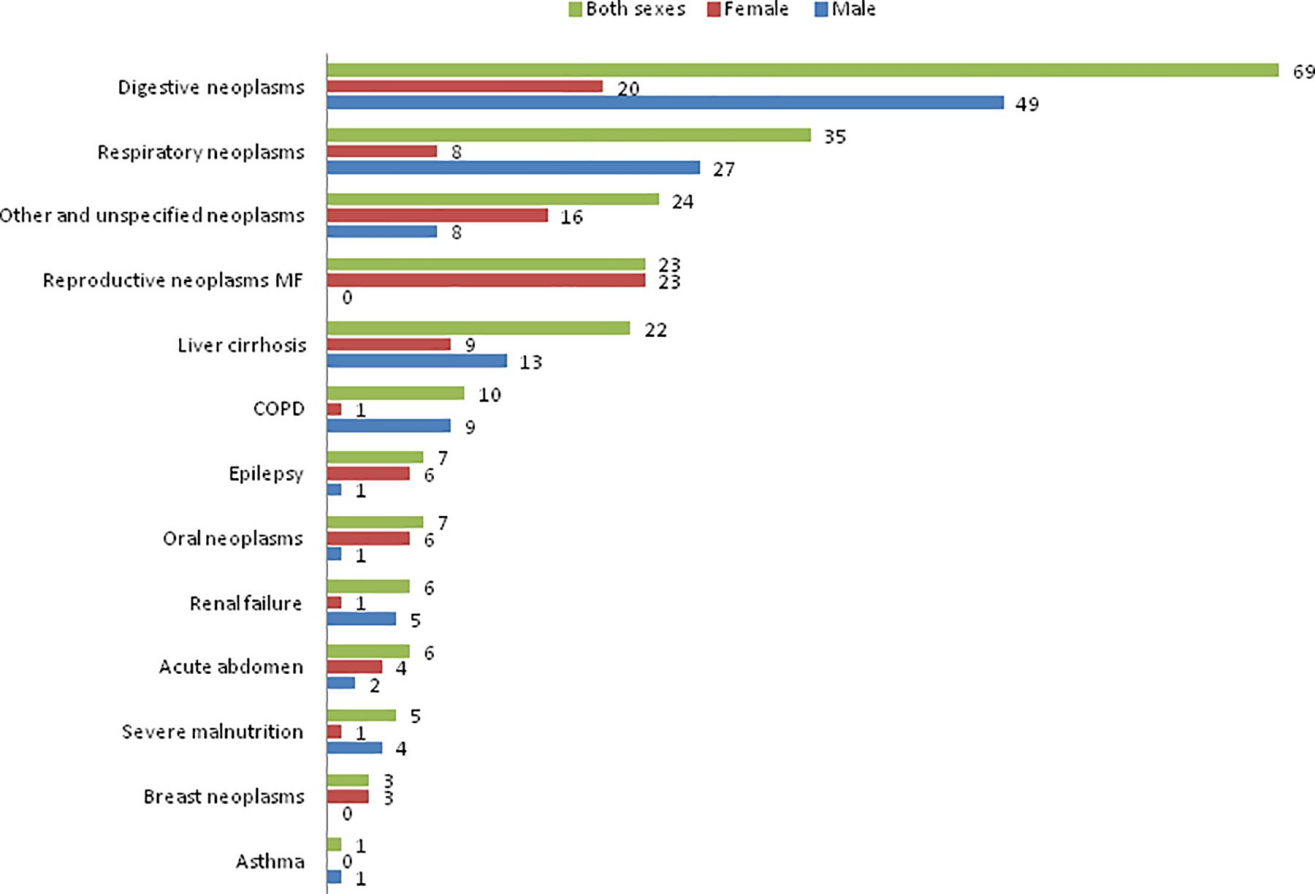
Numbers of deaths from endemic non-communicable diseases, PNGIMR’s CHESS, 2020.

Fig 3 showed the leading CODs from endemic NCDs, with digestive neoplasms as the first leading COD, followed by respiratory neoplasms, and other unspecified neoplasms. The number of male deaths to these cancers was significantly higher than female counterparts (Chi-squared test p-value at 0.012).

### Socioeconomic demographic factors associated with mortality from NCDs

Table 1 reports the distribution of deaths attributed to emerging NCDs, endemic NCDs and other CODs by sociodemographic characteristics of the deceased. The proportions of male and female deaths from emerging NCDs and from endemic NDCs were similar, around 20%-24% of the total deaths. The proportions of deaths from emerging and endemic NCDs increased with age, but the increase in emerging NCD-attributed deaths was most obvious from ages 35–44 (5%) to ages 45–54 years (30%). The increased proportion of deaths from endemic NCDs occurred in a more linear fashion from ages 15–24 through to ages 45–64. Different patterns of mortalities from emerging and endemic NCDs were observed between urban and rural populations, with higher proportions of emerging NCDs related deaths in the former and higher proportions of endemic NCDs related deaths in the latter. Port Moresby had the highest proportions of deaths from both emerging and endemic NCDs, each accounting for 30% of the total deaths. While Madang had a higher proportion of deaths from endemic NCDs than emerging NCDs, 18% compared to 13%, ENB had higher proportion of emerging NCDs deaths than endemic NCDs deaths, 26% versus 18%, respectively. In relation to household wealth quintile, people from the richest households (5^th^ quintile) had the highest proportion of deaths from emerging NCDs (28%) and people from rich households (4^th^ quintile) had the highest proportion of deaths from endemic NCDs (25%).

**Table 1:** Distribution of deaths attributed to emerging NCDs, endemic NCDs and other causes of death by sociodemographic characteristics of the deceased, PNGIMR’s CHESS 2020.

		Emerging NCD attributed CODs	Endemic NCD attributed CODs	Other CODs	All CODs
Sex	Male	124 (24.1%)	116 (22.6%)	274 (53.3%)	514 (100.0%)
	Female	83 (20.1%)	97 (23.5%)	232 (56.3%)	412 (100.0%)
Total		207(22.4%)	213 (23.0%)	506 (54.6%)	926 (100.0%)
Age group	0–4	1(1.5%)	2 (3.0%)	64 (95.5%)	67 (100.0%)
	5–14	2 (7.7%)	2 (7.7%)	22 (84.6%)	26 (100.0%)
	15–24	8 (12.9%)	7 (11.3%)	47 (75.8%)	62 (100.0%)
	25–34	10 (10.1%)	18 (18.2%)	71 (71.7%)	99 (100.0%)
	35–44	5 (5.3%)	22 (23.4%)	67 (71.3%)	94 (100.0%)
	45–54	44 (29.5%)	44 (29.5%)	61 (40.9%)	149 (100.0%)
	55–64	56 (32.9%)	42 (24.7%)	72 (42.4%)	170 (100.0%)
	65–74	47 (32.9%)	48 (33.6%)	48 (33.6%)	143 (100.0%)
	75+	34 (30.1%)	28 (24.8%)	51 (45.1%)	113 (100.0%)
Total		207 (22.4%)	213 (23.1%)	503 (54.5%)	923 (100.0%)
Sector	Urban	56 (24.6%)	48 (21.1%)	124 (54.4%)	228 (100.0%)
	Rural	147 (21.7%)	159 (23.5%)	371 (54.8%)	677 (100.0%)
Total		203 (22.4%)	207 (22.9%)	495 (54.7%)	905 (100.0%)
Province	POM	9 (30.0%)	9 (30.0%)	12 (40.0%)	30 (100.0%)
	Central	66 (22.9%)	65 (22.6%)	157 (54.5%)	288 (100.0%)
	EHP	64 (21.3%)	74 (24.7%)	162 (54.0%)	300 (100.0%)
	Madang	10 (13.2%)	14 (18.4%)	52 (68.4%)	76 (100.0%)
	ESP	28 (24.1%)	30 (25.9%)	58 (50.0%)	116 (100.0%)
	ENB	30 (25.9%)	21 (18.1%)	65 (56.0%)	116 (100.0%)
Total		207 (22.4%)	213 (23.0%)	506 (54.6%)	926 (100.0%)
Household wealth quintiles	Poorest	31 (22.5%)	29 (21.0%)	78 (56.5%)	138 (100.0%)
	Poor	33 (23.9%)	30 (21.7%)	75 (54.3%)	138 (100.0%)
	Middle	30 (21.7%)	32 (23.2%)	76 (55.1%)	138 (100.0%)
	Rich	27 (19.6%)	35 (25.4%)	76 (55.1%)	138 (100.0%)
	Richest	38 (27.7%)	30 (21.9%)	69 (50.4%)	137 (100.0%)
Total		159 (23.1%)	156 (22.6%)	374 (54.3%)	689 (100.0%)

Table 2 shows the sociodemographic factors associated with mortalities from emerging and endemic NCDs. The risk of dying from emerging NCDs was significantly lower among the populations of age groups 5–14, 15–24, 25–34, 35–44, compared with the oldest age group (75+) (p-values < 0.05), meaning the risk of dying from these diseases significantly increased from the ages of 45 years and above. Compared to the population in rural areas, urban populations were nearly twice more likely to die from emerging NCDs (OR: 1.9 [95% CI: 1.1–3.3]; p-value: 0.018). People in Madang were 70% less likely to die from emerging NCDs than those who live in ENB (OR: 0.3 [95% CI: 0.13–0.73]; p-value: 0.007). However, the risk of dying from emerging NCDs was not significantly different between two sexes and household wealth quintiles (p-values >0.05).

**Table 2:** Odds ratios of mortalities attributed to emerging NCDs and endemic NCDs among general population in Papua New Guinea PNGIMR’s CHESS, 2020.

Socioeconomic demographic factor				Emerging NCDs attributed mortality^a^				Endemic NCDs attributed mortality			
	Category	N	%	Odd ratio	Lower bound	Upper bound	P-value	Odds ratio	Lower bound	Upper bound	P-value
Sex	Male	387	58.2%	1.219	0.822	1.807	0.324	0.872	0.571	1.331	0.525
	Female	278	41.8%	Ref.				Ref.			
Age group	0–4	39	5.9%	N/A	N/A	N/A	N/A	0.114	0.014	0.896	0.039
	5–14	20	3.0%	0.137	0.017	1.092	0.060	0.229	0.028	1.847	0.166
	15–24	45	6.8%	0.366	0.142	0.946	0.038	0.393	0.122	1.266	0.117
	25–34	64	9.6%	0.212	0.080	0.563	0.002	0.867	0.374	2.013	0.741
	35–44	62	9.3%	0.140	0.045	0.433	0.001	0.824	0.351	1.934	0.656
	45–54	115	17.3%	0.834	0.447	1.556	0.567	0.890	0.431	1.838	0.753
	55–64	131	19.7%	0.960	0.523	1.763	0.896	0.857	0.420	1.747	0.671
	65–74	102	15.3%	1.202	0.640	2.260	0.567	1.134	0.552	2.330	0.732
	75+	87	13.1%	Ref.				Ref.			
Sector	Urban	226	34.0%	1.922	1.116	3.310	0.018	1.250	0.687	2.275	0.464
	Rural	439	66.0%	Ref.				Ref.			
Province	POM	30	4.5%	1.078	0.409	2.840	0.879	2.394	0.850	6.741	0.098
	Central	167	25.1%	1.635	0.840	3.182	0.148	1.920	0.853	4.322	0.115
	EHP	277	41.7%	0.900	0.508	1.594	0.718	1.814	0.897	3.665	0.097
	Madang	75	11.3%	0.314	0.135	0.730	0.007	1.260	0.516	3.076	0.611
	ENB	116	17.4%	Ref.				Ref.			
Household wealth quintiles	Poorest	135	20.3%	0.773	0.428	1.396	0.394	1.516	0.786	2.927	0.215
	Poor	135	20.3%	0.818	0.457	1.461	0.497	1.264	0.649	2.460	0.491
	Middle	130	19.5%	0.721	0.396	1.314	0.286	0.916	0.447	1.876	0.809
	Rich	130	19.5%	0.571	0.309	1.058	0.075	1.344	0.688	2.626	0.387
	Richest	135	20.3%	Ref.				Ref.			
Total valid		665	100.0%								

^a, b^The reference category is ‘other CODs’.

For endemic NCDs, only children aged from 0–4 years had a significantly lower risk of dying compared with the oldest age group (75+) (OR: 0.1 [95% CI: 0.01–0.9]; p-value: 0.04). The risks of dying from endemic NCDs were not significantly different between sexes, age groups, urban-rural sectors, provinces, and household wealth quintiles.

## Discussion

This study is the first to present an overview of the scope and distribution of sociodemographic factors of mortalities attributed to emerging and endemic NCDs at the population level in PNG. The data show that the diversity of NCD mortalities is occurring across sub-populations. Epidemiological shifts were progressing differently between emerging NCDs and endemic NCDs across ages and sexes, urban and rural areas, provinces, and social classes of the population. The data have indicated an overall trend of increased mortalities associated with emerging NCDs in the urban population, who were twice more likely to die from these diseases than those from rural areas. The risk of dying from emerging NCDs was particularly high among those who aged 45 years and above, and resided in Port Moresby, and urbane areas of Central, Eastern Highlands, East Sepik, and East New Britain provinces. By contrast, the association of the endemic NCDs mortality with sociodemographic characteristics of the deceased was unclear, except for children under five years of age, who were less likely to die from these diseases because these chronic conditions often commence in later years of life and last for years before the patients died from it.

About 85% of the PNG population reside in rural areas, mostly involving in subsistence-based agriculture [29, 30]. The urban surveillance sites in the catchment of CHESS can be basically classified into three types based on their level of urbanisation: (i) Towns in low level of urbanisation are more associated with household economy with incomes mostly from the agricultural industries such as farming and fishery i.e. Goroka in EHP and Maprik in East Sepik provinces; (ii) Towns in middle level of urbanisation are more associated with the development of the private sector with waged employment mostly from mining and food processing industries i.e. Hiri in Central, Newtown in Madang and Kokopo in ENB provinces; and (iii) Towns with high level of urbanisation are more associated with the development of the public sector and services i.e. Hohola in POM [23].

Urbanisation appears a major socioeconomic determinant of the mortality transition in PNG, shifting the CODs in the population from infectious diseases towards NCDs. Unlike the urbanisation processes in other LMICs where the epidemiological transition from infectious diseases to NCDs progress slowly over a period of time [30], this study shows evidence suggesting that the mortality transition in PNG had taken place at an early stage of the urbanisation. Given the recent urbanisation progress in PNG, the epidemiological transition has already had an impact with an increase in mortality attributed to emerging NCDs among the urban population.

The physical and human contrasts, extensive swamps, impenetrable bush-tangled rocky terrain, and high mountains are still effective barriers to human mobility, settlement and communication in PNG [31]. However, PNG people are becoming more mobile, even in remote areas of the country, and migration destinations are most likely associated with job and employment opportunities. Along with the expansion of urban areas and the development of new towns, farmers are leaving their homes in rural areas to move into urban centres, resulting in increased population growth across cities and towns in PNG. The internal migration flow was slow and small at the beginning, but has increased more recently in areas, where large development projects are taking place, attracting internal migration flows into and out of the project sites. The crude gross migration rate was as high as 16% across the surveillance sites in 2018 [21].

Urbanization interplays with local contextual factors providing possible explanations for the variation and differences in trends in mortality in PNG. The burden of NCDs is not equally distributed across the provinces. POM had the highest proportion of mortalities from emerging and endemic NCDs, accounting for 60% of the total deaths, followed by Central province (45%). Central Province has experienced a rapid urbanisation in the 2010s with the development of the PNG Natural Liquefied Project. Urban populations have been reportedly moving away from traditional foods, diets, and life-styles [32]. In new resettlement areas, health risks are reportedly associated with urban poverty, including poor housing conditions [24], unhealthy lifestyles and eating behaviours, together with social stresses associated with the working environment and social conflicts [33]. The increase of cash flow and the local economic growth are thought to contribute to increased access to and consumption of processed foods [32, 34]. Children in these sites were reported as consuming high levels of sugar, soft drinks and salty fast foods [35]. Half of the adult population and 25% of adult patients in Central Province were identified as overweight or obese. Nearly half of male adults smoked tobacco. NCDs such as acute cardiac diseases, stroke and diabetes have emerged among other NCDs as a result of the high-risk local environment [14].

Compared to mortality transitions in other countries in the South Pacific Region, the mortality transition in PNG is likely slower. With about 45% of deaths due to emerging and endemic NCDs, our finding is similar to those reported in the recent study in PNG (48.8%), lower than Solomon (70%) [19], and Fiji (80%) [36]. Our data support that emerging NCDs such as cardiovascular diseases, stroke, diabetes, and endemic NCDs including cancers and chronic respiratory diseases are among the leading CODs in the population, with the highest impact on socioeconomic development and healthcare systems in LMICs [37, 38], where weak governance, poor administration, and fragile health systems with limited human and financial resources are common reasons for ineffective responses to planning and management of urban health [39].

### Limitations

Mortality data from VA interviews were used to assign possible CODs, but the information about the deaths of the deceased provided by the relatives could be biased, particularly for those interviews which were conducted two or more years after the date of death [40]. The deaths in the communities were identified by the village-based data reporters, but these data were not included unless the deaths occurred within the catchment areas of the surveillance system during the data collection period. Our field work experiences and mortality surveillance records suggest that about 20% of deaths in the urbane sites in POM and Madang and about 15% of deaths in the rural sites in Eastern Highlands, East New Britain, East Sepik, and Central provinces occurred in health facilities [13, 18].

Since CHESS was designed to have a primary health facility included within the catchment areas of each surveillance site, 95% of the surveillance population reported having access to primary healthcare services [25]. About 90% of the population of working age (15–64 years) reported having some kind of jobs in the past two weeks and the education attainment was low (net enrollment rate in primary education below 60%) [41]. This could be a reason for why those variables on women’s access to health services, employment and education were non-significant in the statistical modeling.

## Conclusion

The study has provided insights into the current mortality transition in PNG. Urbanisation and local contextual factors could be the key socioeconomic correlates of the mortality transition in PNG. The study found that different sociodemographic factors contributed to the increased mortalities from emerging and endemic NCDs. Different health policies and interventions are needed to target urban and rural populations to change unhealthy life styles to reduce premature mortality from NCDs. The increased risk of dying from emerging NCDs such as cardiovascular diseases, stroke and diabetes mellitus is evident in urban areas. Urban residents are twice more likely to die from these emerging NCDs, particularly among those who are 45 years and older, and from provinces undergoing rapid urbanisation such as Port Moresby and Central province. By contrast, mortality attributed to endemic NCDs such as neoplasms, chronic respiratory diseases and malnutrition are evenly distributed across ages, sexes, urban-rural sectors, provinces and social classes.

As urbanisation may continues in PNG in coming years, profound demographic changes including domestic migration will have greater impacts on the health and well-being of the population. Policy level changes are needed to reduce risk factors among the most at risk populations. This study has provided evidence that can be used by PNG Government agencies and the health sector. Health systems are much in need to reform and restructure to effectively respond to new challenges and to meet with the increased demand for healthcare services for NCDs among the population. Equitable access to effective and quality preventive measures and curative services are needed to protect the population from premature deaths from NCDs. NCDs prevention programmes and interventions need to focus on emerging NCDs and target populations in urban areas and in provinces, where urbanisation is occurring at a rapid pace.

Socioeconomic development programmes and health interventions are needed to alleviate poor health conditions, change unhealthy lifestyles and eating behaviors, and reduce the social stress of urban living. Social planners, public health experts and stakeholders need to make greater efforts to improve the health and well-being of the population in an equitable, effective and sustainable manner. This calls for more data and analysis of causes of death to assist the PNG Government in developing strategies to effectively address multiple complex public health issues in such critical epidemiological transitional period in PNG.

## Supporting information

S1 Data Dataset of NCD attributed mortality in Papua New Guinea, PNGIMR’s CHESS, 2020. (SAV)
